# The Geography of Diabetes by Census Tract in a Large Sample of Insured Adults in King County, Washington, 2005–2006

**DOI:** 10.5888/pcd11.140135

**Published:** 2014-07-24

**Authors:** Adam Drewnowski, Colin D. Rehm, Anne V. Moudon, David Arterburn

**Affiliations:** Author Affiliations: Colin D. Rehm, Center for Public Health Nutrition, University of Washington, Seattle, Washington; Anne V. Moudon, College of Built Environments, University of Washington, Seattle, Washington; David Arterburn, Group Health Research Institute, Seattle, Washington.

## Abstract

**Introduction:**

Identifying areas of high diabetes prevalence can have an impact on public health prevention and intervention programs. Local health practitioners and public health agencies lack small-area data on obesity and diabetes.

**Methods:**

Clinical data from the Group Health Cooperative health care system were used to estimate diabetes prevalence among 59,767 adults by census tract. Area-based measures of socioeconomic status and the Modified Retail Food Environment Index were obtained at the census-tract level in King County, Washington. Spatial analyses and regression models were used to assess the relationship between census tract–level diabetes and area-based socioeconomic status and food environment variables. The mediating effect of obesity on the geographic distribution of diabetes was also examined.

**Results:**

In this population of insured adults, diabetes was concentrated in south and southeast King County, with smoothed diabetes prevalence ranging from 6.9% to 21.2%. In spatial regression models, home value and college education were more strongly associated with diabetes than was household income. For each 50% increase in median home value, diabetes prevalence was 1.2 percentage points lower. The Modified Retail Food Environment Index was not related to diabetes at the census-tract level. The observed associations between area-based socioeconomic status and diabetes were largely mediated by obesity (home value, 58%; education, 47%).

**Conclusion:**

The observed geographic disparities in diabetes among insured adults by census tract point to the importance of area socioeconomic status. Small-area studies can help health professionals design community-based programs for diabetes prevention and control.

## Introduction

Identifying areas of high diabetes prevalence can have an impact on public health prevention and intervention programs. First, an elevated risk of diabetes incidence is associated with low socioeconomic status (SES) (1). Second, studies based on Centers for Disease Control and Prevention (CDC) estimates of diagnosed diabetes prevalence at the county level link high diabetes risk with obesity and sedentary lifestyles (2). Those county-level studies helped to identify a geographic region of the United States, mostly in southern states, as the diabetes belt (3).

Geographic disparities in diabetes prevalence at both the state and county levels have been observed in national health surveys, such as the Behavioral Risk Factor Surveillance System (BRFSS) (3–6). Geographic variations in diabetes prevalence were also found in metropolitan statistical areas (7), ZIP code areas (8), and census tracts in Canada (9). However, even more detailed data are needed to document disease burden and its associated health care costs at the local level. Such estimates are beyond the scope of CDC county-level estimates or national BRFSS surveys that are typically coded at the county and ZIP code levels only.

Small-area estimates, often based on modeling, are useful to local health agencies for targeting scarce resources toward areas with greatest need. The prevalence of heart disease in ZIP Code Tabulation Areas (ZCTAs) were predicted by using multilevel small-area estimate modeling that linked BRFSS data with county-level covariates (10). More recently, childhood obesity prevalence at the census block–group level was predicted by using geocoded data from a national survey and multilevel modeling (11). Our study used clinical data to examine the geographic distribution of diabetes at the census-tract level in King County, Washington. Census tracts are small areas that have an average population of 4,000, are smaller than ZCTAs, and are larger than census block groups.

Using geo-localized health and anthropometric data from insurance companies for small-area studies is a new direction in diabetes and obesity research. The objective of our study was to explore spatial relationships between area-based measures of SES, obesity, and diabetes for subcounty areas to arrive at a better understanding of the impact of local environmental factors on disease rates. 

## Methods

### Study population

We obtained data from the Group Health Cooperative (Group Health) health care system, which manages care and coverage for more than 600,000 residents of Washington State. Of Group Health adult members (aged ≥18 y) residing in King County during the 2 years from January 2005 through December 2006, 96.5% (n = 59,767) were geocoded to census tracts. Diabetes status was determined by using laboratory, pharmacy, and diagnosis data from Group Health’s electronic medical record databases. An individual having any of the following was defined as having diabetes: 1) more than 1 fill for diabetes-specific medication; 2) an HbA1c of 7.0% or more on 1 or more occasions (12); 3) a fasting blood glucose of 126 mg/dL or more on 1 or more occasions; 4) a random blood glucose of 200 mg/dL or more on 1 or more occasions; or 5) 1 or more outpatient or inpatient *International Classification of Diseases, Ninth Revision* (ICD-9) codes (250.x) related to diabetes. Because the ICD-9 codes for diabetes do not specify type of diabetes (ie, type 1 or type 2), our cohort included patients with both types, although nationally 90% to 95% of diabetes is type 2 (13). Heights and weights recorded during routine primary care visits to Group Health clinics were used to calculate body mass index (BMI). Individuals with a BMI of 30 kg/m^2^ or more were classified as obese.

### Diabetes prevalence

The use of an empirical Bayes (EB) tool to estimate the smoothed diabetes prevalence (14) allowed us to include census tracts with small sample sizes that would otherwise have to be excluded. The tool is used to drive prevalence estimates with extreme values or those based on small sample sizes toward the countywide prevalence. We used a spatially naïve EB tool, which did not use information from nearby census tracts to smooth diabetes prevalence.

We used a modified Queen’s contiguity matrix, which defines neighboring census tracts as those sharing a border or corner. The default matrix was modified to include bodies of water and transportation arteries such as bridges. Spatial weights were modified to reflect these relationships.

### Geographic areas

Area-based SES measures from the 2000 Census were percentage of adults with a college degree, median household income, and median home value. Home values were transformed by taking the natural logarithm (*ln*), and effect sizes were estimated on the basis of a 50% increase. Additional census covariates were percentage of non-Hispanic black residents, percentage of Hispanic residents, and population density (population per square mile excluding major water features). Data on mean age of individuals in each census tract were obtained from Group Health.

Secondary analyses explored the potential role of the food environment in explaining the association between SES variables and diabetes prevalence. Data for the CDC’s Modified Retail Food Environment Index (mRFEI) were obtained for King County at the census-tract level (15). The mRFEI is a ratio-based measure of healthful-to-total food retailers, with higher values indicating a more healthful food environment (15). The CDC defined supermarkets, large grocery stores, fruit and vegetable markets, and warehouse clubs as healthful sources and fast food restaurants and convenience stores as less healthful sources.

### Spatial model development and diabetes estimation

The Moran’s *I* statistic tested whether the geographic distribution of diabetes was spatially random across King County (16). A Moran’s *I* value of 1.0 denotes perfect spatial autocorrelation, 0 denotes a random spatial pattern, and −1.0 indicates complete spatial dispersion. Given indications of spatial patterning, we further evaluated local clustering by using the Getis-Ord Gi* statistic (17). Significance of local clustering was based on a *P* of less than .05 and 99,999 Monte Carlo simulations.

The association between smoothed diabetes prevalence and each SES variable was then modeled individually. For none of the predictors or their covariates did the variance-inflation factor exceed 1.8, which indicated a lack of colinearity between predictors and covariates. For each SES variable, 4 models were fit. Model 1 adjusted for age; Model 2 adjusted for age, race/ethnicity, and population density; Model 3 adjusted for all factors above and mRFEI; Model 4 adjusted for all factors above and obesity prevalence. Models 3 and 4 assessed the extent to which associations between SES and diabetes could be explained by obesity or by the food environment (mRFEI).

We then evaluated spatially autoregressive linear regression models. The spatial-error regression model accounts for spatial dependence of the measured variables and unknown or poorly measured predictors that have a spatial component. The spatial-error model partially accounts for similarities in the built environment in contiguous areas that are not fully accounted for by population density or by demographic covariates. The Akaike information criterion (AIC) was used to determine the best-fitting model. Finally, we evaluated the residual spatial dependence in the distribution of diagnosed diabetes by estimating Moran’s *I* statistic for the residuals from a series of models accounting for an increasing number of covariates, including age, race/ethnicity, population density, and area-based socioeconomic variables.

Both mRFEI and obesity were considered potential mediators of the SES–diabetes association. We used a casual mediation framework to estimate the proportion of the association between SES and diabetes that was explained by each variable (18). The primary mediation analyses were based on the spatially naïve models. The R package (The R Project for Statistical Computing, Institute for Statistics and Computing, Vienna, Austria) was used to conduct the mediation analysis and to estimate confidence intervals (CIs) of the proportion explained on the basis of 100,000 simulations.

Statistical and spatial analyses were performed using OpenGeoDa (GeoDa Center for Geospatial Analysis and Computation and Arizona Board of Regents, Tempe, Arizona), Stata 13.1 (StataCorp LP, College Station, Texas) and the R mediation package (19). Mapping was done by using ArcGIS 10.2 (Esri, Redlands, California).

## Results

The final analysis was based on 371 census tracts and 59,767 insured adults. The number of participants per census tract ranged from 22 to 413; the average was 161. Of the sample, 22% were aged 55 to 64, and 30% were aged 65 or older. By comparison, 13% of the King County population were aged 55 to 64, and 13% were aged 65 or older. The crude prevalence of diabetes was 12.7% (men, 15.4%; women, 10.9%). The unsmoothed prevalence of diabetes ranged from 4.0% to 42.1%. EB-smoothed prevalence of diabetes ranged from 6.9% to 21.2%.

**Figure 1:**
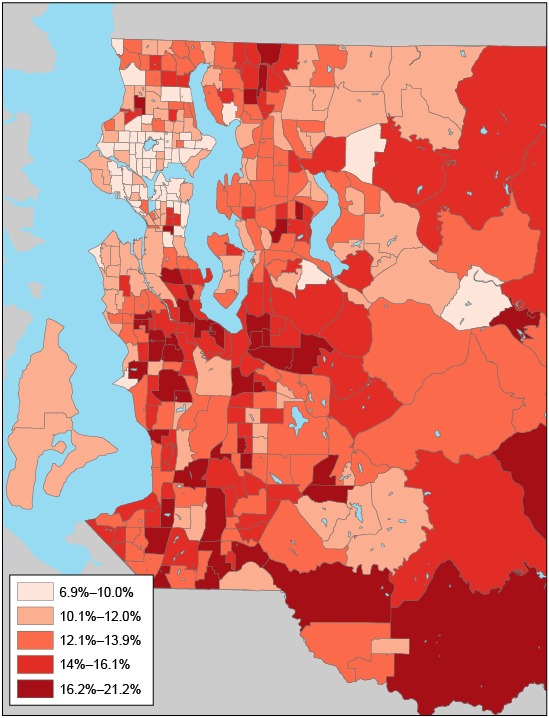
Diabetes prevalence was smoothed by using an empirical Bayes tool, King County, Washington, 2005–2006. Eastern portions of census tracts in eastern King County are not shown because they are sparsely populated and consist mostly of forested land.

**Figure 2:**
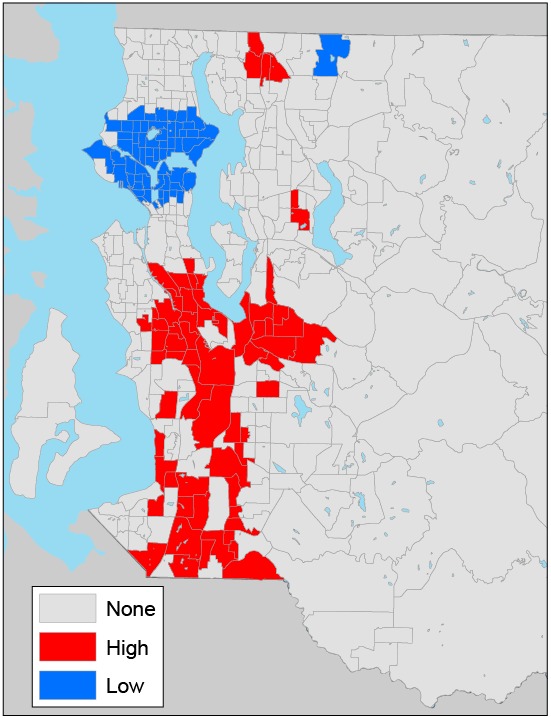
Local clusters of census-tract–level diabetes prevalence in King County, Washington, 2005–2006, as determined by the Getis-Ord* (Gi*) statistic (17). Eastern portions of census tracts in eastern King County are not shown because they are sparsely populated and consist mostly of forested land. In the key, “none” indicates no clustering; “high,” a cluster of census tracts that have a high prevalence of diabetes; “low,” a cluster of census tracts that have a low prevalence of diabetes.

Census-tract–level prevalence of diabetes showed a nonrandom spatial pattern (Figure 1 and Figure 2); census tracts with a high prevalence of diabetes tended to cluster together (Moran’s *I* = 0.46, *P* < .001) (Table 1). High prevalence of diabetes was concentrated in south and southeast King County (Figure 2). We found significant spatial autocorrelations for the SES variables of interest. Census tracts with a high median household income, a high percentage of adults with a college degree, a high median home value, a high percentage of black residents, a high percentage of Hispanic residents, and high population density tended to cluster together. The extent of spatial clustering was particularly strong for the percentage of adults with a college degree (Moran’s *I* = 0.81) and the percentage of black residents (Moran’s *I* = 0.74) (Table 1).

**Table 1:** Univariate Moran’s *I* Statistic and Bivariate Association of Variables With Smoothed Prevalence Of Diabetes Among 59,767 Group Health Members in King County, Washington, 2005–2006

Variable	Moran’s *I* Statistic^a^^,^ b (95% CI)	Correlation Coefficient (95% CI)
Smoothed prevalence of diabetes	0.46 (0.40–0.52)	—
Crude prevalence of diabetes	0.36 (0.30–0.42)	—
Smoothed prevalence of obesity	0.67 (0.61–0.72)	0.58 (0.51 to 0.65)
Natural logarithm for median home value	0.65 (0.60–0.71)	−0.49 (−0.41 to −0.56)
Percentage of residents with a college degree	0.81 (0.77–0.85)	−0.60 (−0.53 to −0.66)
Median household income	0.61 (0.55–0.66)	−0.18 (−0.08 to −0.28)
Percentage of black residents	0.74 (0.69–0.78)	0.18 (0.08 to 0.28)
Percentage of Hispanic residents	0.43 (0.37–0.47)	0.28 (0.19 to 0.38)
Population per square mile	0.58 (0.53–0.63)	−0.30 (−0.39 to −0.21)
mRFEI	0.23 (0.17–0.28)	−0.03 (−0.13 to 0.08)

Abbreviations: CI, confidence interval; —, does not apply; mRFEI, Modified Retail Food Environment Index.

a Higher values for Moran’s *I* statistic indicate greater extent of spatial clustering for that variable. A value of 0 indicates a random spatial pattern, a value of −1 indicates perfect dispersion, and a value of 1 indicates perfect clustering.

b *P* value for all Moran’s *I* statistics < .001

**Supplemental Figure 1:**
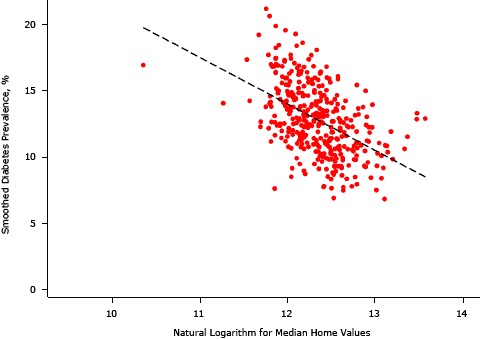
Association between smoothed diabetes prevalence and the natural logarithm of median home value, King County, Washington, 2005–2006.

**Supplemental Figure 2:**
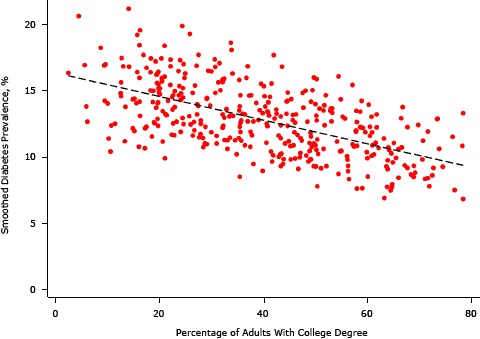
Association between smoothed diabetes prevalence and the percentage of residents with a college degree, King County, Washington, 2005–2006.

**Supplemental Figure 3:**
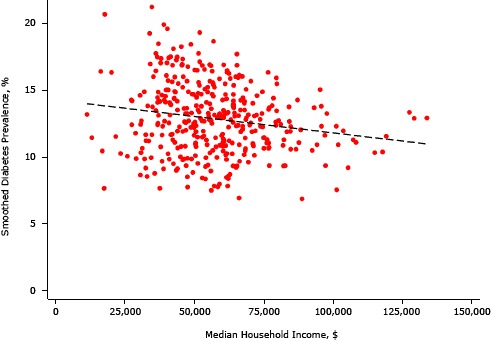
Association between smoothed diabetes prevalence and median household income, King County, Washington, 2005–2006.

**Supplemental Figure 4:**
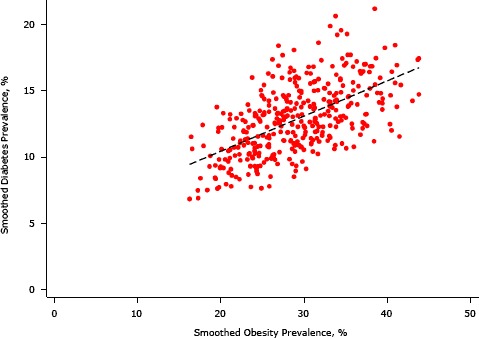
Association between smoothed diabetes prevalence and smoothed obesity prevalence, King County, Washington, 2005–2006.

In bivariate analyses, each area SES variable was significantly associated with an EB-smoothed prevalence of diabetes; we found the strongest negative association between the percentage of residents with a college degree and median home value (Table 1, Table 2, and Supplemental Figures 1–4). Percentages of non-Hispanic black and Hispanic residents were positively associated with the prevalence of diabetes, and high population density was negatively associated. 

**Table 2:** Ordinary Least Squares and Spatial Regression Models for Relationship Between Area-Based Measures of Socioeconomic Status and Smoothed Diabetes Prevalence Among 59,767 Group Health Members in King County, Washington, 2005–2006

	*Ln* Median Home Value^a^ (per 50% Increase)		% of Residents With a College Degree (per 10% Increase)		Median Household Income (per $10,000 Increase)	
	β (95% CI)	AIC^b^	β (95% CI)	AIC^b^	β (95% CI)	AIC^b^
**Age-adjusted**						
OLS model	−1.6 (−1.9 to −1.4)	−1,787	−0.9 (−1.1 to −0.8)	−1,848	−0.3 (−0.4 to −0.2)	−1,668
Spatial error model	−1.2 (−1.5 to −0.9)	−1,845	−0.9 (−1.1 to −0.8)	−1,885	−0.4 (−0.5 to −0.2)	−1,820
**Model 1^c^**						
OLS model	−1.4 (−1.7 to −1.1)	−1,830	−0.9 (−1.0 to −0.7)	−1,860	−0.3 (−0.5 to −0.1)	−1,754
Spatial-error model	−1.2 (−1.6 to −0.9)	−1,856	−0.9 (−1.1 to −0.7)	−1,886	−0.3 (−0.5 to −0.2)	−1,825
**Model 2^d^**						
OLS model	−0.6 (−0.9 to −0.2)	−1,878	−0.4 (−0.6 to −0.1)	−1,874	−0.03 (−0.2 to 0.1)	−1,867
Spatial error model	−0.6 (−0.9 to −0.2)	−1,897	−0.4 (−0.7 to −0.1)	−1,893	−0.11 (−0.3 to 0)	−1,888
Percentage mediated by obesity– OLS^c^	58% (41% to 81%)	—e	47% (29% to 67%)	—e	90% (56% to 184%)	—e

Abbreviations: *Ln*, natural logarithm; CI, confidence interval; AIC, Akaike information criterion; OLS, ordinary least squares.

a Median home values were analyzed on the natural logarithm scale and can be interpreted as a relative increase of 50% (eg, from $200,000 to $300,000 or $600,000 to $900,000).

b For AIC, lower values indicate better model fit. An evaluation of the Bayesian information criteria resulted in an identical ranking of models. Model 2 spatial-error model was the most informative model for each area-based SES variable of interest.

c Adjusted for age, percentage of black residents, percentage of Hispanic residents, and population density.

d Adjusted for model 1 + smoothed prevalence of obesity.

e AIC not estimated for mediation models.

The spatial-error model showed that when holding other factors constant, for each 50% increase in median home value (eg, from $200,000 to $300,000), the prevalence of diabetes was 1.2 percentage points lower (95% CI, −1.6 percentage points to −0.9 percentage points) (Table 2). For each 10% increase in the percentage of adults with a college degree, the prevalence of diabetes was 0.9 percentage points lower (95% CI, −1.1 percentage points to −0.7 percentage points). For each $10,000 increase in median household income, the prevalence of diabetes was 0.3 percentage points lower (95% CI, −0.5 percentage points to −0.2 percentage points). The spatial dependence of diabetes data was fully accounted for by area-SES variables. We no longer found positive spatial autocorrelation after accounting for the covariates and the spatial dependence of the data (Moran’s *I* statistic between −0.05 and 0 for each model).

The association between area SES and diabetes was mediated to a large extent by the prevalence of obesity (Table 2). For the association between median home value and diabetes prevalence, 58% (95% CI, 41%–81%) was explained by differences in obesity prevalence after adjusting for age, race/ethnicity, and population density. For the association between diabetes prevalence and percentage of adults with a college degree, 47% (95% CI, 29%–67%) was explained by obesity. Similar effects were found in models that adjusted only for age.

In bivariate analyses, 24% of the variance in diabetes prevalence was explained by median home value. Adding population density, race/ethnicity, age, and spatial dependence to the model increased the variance explained to 48%. Similarly, 45% of the variance in diabetes prevalence was explained by college education; 52% was explained after adjusting for covariates. The 3 area-SES measures accounted for 39% of the variance in diabetes prevalence.

An examination of crude and adjusted linear regression models with standardized β-coefficients confirmed that college education had the strongest negative association with diabetes, followed by property value and median household income. By contrast, the principal metric of the food environment, the mRFEI, was not associated with diabetes prevalence either independently or after accounting for demographic covariates. In models adjusted for age, race/ethnicity, and population density, none of the SES measures were associated with the mRFEI. The mRFEI was not a mediator of the association between area-SES measures and diabetes prevalence.

An evaluation of the Moran’s *I* statistic for the residuals showed that much of the spatial dependence of diabetes was accounted for by area-SES variables (Table 3). We found strong spatial clustering in crude, age-adjusted, and age/race-adjusted models. Adding population density reduced the strength of clustering. Adding home value and college education variables further reduced clustering, although modest residual spatial dependence remained.

**Table 3:** Effect of Covariates in Accounting for Spatial Clustering of Diabetes Prevalence Among 59,767 Group Health Members in King County, Washington, 2005–2006

Model	Moran’s *I* Statistic of Residuals (95% CI)^a^	*P* Value Compared With Crude	*P* Value Compared With Model 3
Crude (intercept-only model)	0.46 (0.40–0.52)	Reference	<.001
Model 1: Age-adjusted	0.48 (0.43–0.54)	.54	<.001
Model 2: Model 1 + race/ethnicity	0.40 (0.34–0.46)	.19	.001
Model 3: Model 1 + population density	0.27 (0.21–0.32)	<.001	Reference
Model 4: Model 3 + income	0.27 (0.21–0.32)	<.001	.99
Model 5: Model 3 + home value	0.17 (0.11–0.22)	<.001	.02
Model 6: Model 3 + college	0.18 (0.13–0.23)	<.001	.03
Model 7 – Model 6 + income + home value	0.12 (0.07–0.18)	<.001	<.001

Abbreviation: CI, confidence interval.

a Higher values for Moran’s *I* statistic indicate greater extent of spatial clustering of diabetes prevalence. A value of 0 indicates a random spatial pattern, a value of −1 indicates perfect dispersion, and a value of 1 indicates perfect clustering.

## Discussion

We used data from the Group Health Cooperative health care system to conduct spatial analyses of diabetes prevalence at the census-tract level in King County, Washington. Although the estimated prevalence of self-reported diabetes from the 2006–2010 BRFSS in King County was 6% (20), our estimates among insured adults ranged from 6.9% to 21.2%, depending on geographic location. The geographic distribution of diabetes was nonrandom, showing a high concentration in south and southeast King County. This “diabetes corridor” is a microscale version of the diabetes belt in the southeastern United States, consisting of 644 counties in 15 states (2).

Our ecological analyses thus support and extend previous research on the geographic distribution of adult diabetes and its SES correlates in the United States. In many such studies, in which data were aggregated to the county level, obesity and diabetes were regionally concentrated and associated with low levels of education and unemployment (21). However, county-level data are inadequate for health planning purposes; more granular data are required. Although King County is considered one of the healthiest counties in Washington State (22), we observed a smoothed diabetes prevalence of more than 15% in 78 of 371 (21%) of census tracts. By contrast, the county-level estimate of diabetes prevalence, according to BRFSS self-reports, is approximately 6% (20).

Studies have emphasized the contribution of local geographic factors to obesity and diabetes prevalence (23,24). Although state- and county-level maps of obesity and diabetes are best known (25), local health authorities have turned to smaller-area studies. Public Health–Seattle & King County analyzed BRFSS data by 26 health reporting areas (ie, aggregations of ZIP codes) to determine whether obesity and diabetes are concentrated in disadvantaged areas. Our census-tract–level analyses showed that diagnosed diabetes was strongly linked to area SES and in particular to the objective measure of residential property value. By contrast, the spatial clustering of diabetes was not related to the mRFEI, a widely available measure of the food environment.

In previous studies, we established an association between individual residential property values obtained directly from the county tax assessor and self-rated health: the higher the property value, the lower the likelihood of reporting fair or poor health (26) and the lower the prevalence of obesity among women (27). Analyses in adjacent Snohomish County showed a similar relationship between the prevalence of diabetes and home values, using data from BRFSS (28). For most Americans, home equity is among the most important components of accumulated wealth, suggesting the potential utility of this measure in future studies (29).

Census-tract–level maps of obesity and diabetes may provide unique insights into the social and environmental determinants of these conditions. The causal relationship between obesity and type 2 diabetes is thought to be mediated by weight-related changes in insulin resistance. Although a complex interaction among many genes and environmental factors may be involved, we found that the ecologic relationship between SES and adult diabetes was mediated in large part by obesity. These findings have policy implications for obesity-focused interventions to prevent diabetes, with efforts targeted toward neighborhoods and communities at risk.

Our study has numerous strengths. First, height and weight were measured during a clinic visit, not through self-report. Similarly, diabetes prevalence was estimated on the basis of medical records, rather than self-report. Third, access to clinical data from health insurers for a large and geographically diverse sample of nearly 60,000 adults allowed us to estimate the prevalence of diabetes at the census-tract level. To our knowledge, census-tract–level estimates of diabetes prevalence have not been published in the United States.

This study has several limitations. First, it was based on a sample of insured persons. Uninsured and Medicaid participants were excluded or underrepresented. The insured population, whether working or retired, may be in better health than the general population of King County. If so, the true disparities in diabetes prevalence by SES may be even more dramatic than those observed here. Second, the data on height and weight may have been subject to bias. Group Health members for whom data on height and weights were available tended to be older, had a higher RxRisk score (30), and were more likely to be women. Ecologic analyses, even at the census-tract level, do not allow for examination of cross-level effects. The paucity of individual-level measures of SES, race/ethnicity, or other factors was an additional limitation that precluded a multilevel analysis. Last, the mRFEI metric may not be the best summary measure of the availability of healthful or unhealthful food. In addition, the association between the food environment and disease in one place does not preclude the observations of different associations in other places.

Nonetheless, our data show that the prevalence of diabetes among insured adults can be reliably predicted by small-area SES, particularly education level and home value. Together, the area-based measures of median household income, median home value, and percentage of residents with a college education accounted for 39% of the variance. Further studies should evaluate these measures in predicting area-level diabetes; such evaluations could result in the creation of a powerful tool for policy making and assessment in public health. The use of health data from insurers to map the geographic distribution of diabetes could be a particularly valuable tool.
